# Philadelphia Hospital

**Published:** 1843-04-29

**Authors:** M. W. Wilson

**Affiliations:** Late Resident Physician of the Philadelphia Hospital,—now of Schenectady, New York


### CLINICAL LECTURES AND REPORTS.

### PHILADELPHIA HOSPITAL.

### CASE OF PHTHISIS PULMONALIS,

*In which the humming sound was very distinct in the jugular veins—Necroscopy—Numerous lesions—Extra-uterine fœtation, &c.*

BY M. W. WILSON, M. D.

Late Resident Physician of the Philadelphia Hospital,—now of Schenectady, New York.

[Communicated by Professor DUNGLISON.]

H. P——, a coloured woman, of middle age, entered the hospital in March, 1842, labouring under phthisis pulmonalis. No anterior history of any interest was obtained from her. She had had a troublesome cough and difficulty of breathing for several years, which had of late become more severe. No treatment was directed, except a simple pectoral drink; and the strength was supported by good air and nourishing diet.

She remained in very much the state in which she entered until May 31st, when she was examined by Dr. Pennock, and the following note was taken.

Decubitus half recumbent; extreme emaciation; pulse small, regular, and slightly corded, 114 in the minute. Percussion obscurely resonant on the right side anteriorly, except just beneath the clavicle, where it is resonant. It is also obscure in the middle portion of the left side, and resonant elsewhere, except in the præcordial space. Posteriorly, the left side is more developed than the right. Percussion flat in the upper two-thirds of the left. Flat also at the summit of the right, and obscure elsewhere. Respiration bronchial in the lower part of the right lung, both anteriorly and posteriorly, and rudely vesicular at the summit. In the left lung, very strong cavernous respiration is heard from the clavicle to the third rib anteriorly, and at the summit posteriorly; bronchial respiration with crepitant rhonchus in the middle two-thirds.

The size of the heart cannot well be pointed out by percussion, owing to the solidification of the left lung.

*Sounds of the heart.*—Between the fifth and sixth ribs, one inch to the left of the left nipple, both sounds are heard, the first blowing. When excited, this blowing is converted into a rasp, and the second sound is lost.

Over the aortic valves both sounds are heard—the first blowing, and the second clear and ringing. The sounds become more rough as we ascend the aorta, until a complete saw sound is heard. The rasping sound is much louder immediately above the sternum in the neck, than it is over the aortic valves. On the right side the sounds are natural.

A strong impulsatory movement is observed immediately to the right of the clavicular portion of the sterno-cleido-mastoid muscle, at which spot a continued humming sound is ordinarily heard, varying in intensity from a drone to a rasp, and which—when raised to this pitch—is divisible into two sounds. This sound is not heard over the subclavian artery, between the scaleni muscles, over the carotid, or over the arch of the aorta. The same sound is heard (drone or hum) under precisely the same circumstances, and in the same position, over the left side.

The patient became more and more emaciated until her death, which occurred in June last. The humming sound, as well as the other facts noted above, continued to be observed.

*Necroscopy about eighteen hours after death.*

A careful dissection of the neck was made with the purpose of ascertaining the relative size and situation of the bloodvessels, in regard to the surrounding parts. The only thing differing from the natural state that was detected, was an enlargement of two of the bronchial glands on the left side. The larger measured eight lines in length, six in breadth, and four in thickness. The other was about one-third of this size. These glands pressed on the internal jugular vein at its junction with the lingual vein. The vein itself was almost entirely devoid of blood. On the right side an enlarged lymphatic gland was found pressing on the transverse vein, at its junction with the vena cava descendens. This gland was about three times the usual size.

The pericardium contained about the ordinary amount of serum, of a deep red colour. It was adherent to the pleura to such an extent, that great difficulty was found in separating them.

The heart measured in circumference, at its base, eight and a half inches, and in length five inches. It was very flabby, and softened. The aortic valves were slightly thickened with intra-membranous depositions; but they were quite flexible. The mitral valve was nearly natural. The right ventricle contained about an ounce and a half of fluid blood, some of which was partly coagulated, and all very tenacious. In the left ventricle there was a small, very firm coagulum.

The pleura costalis and pleura pulmonalis were adherent throughout, on both sides of the chest.

The left lung was almost entirely destroyed. A cavity occupied the upper part: and in the remainder the pulmonary structure was converted into tubercular degeneration, interspersed with small cavities. The summit of the right lung crepitated, under pressure, very feebly. The lower part was almost entirely destroyed by tubercular degeneration.

The liver was of dark colour, and soft; about the normal size, and cirrhosed. The stomach was healthy.

The tubercular portion of the kidneys was of a dark red colour. The cortical of a dusky yellow, with granular degeneration.

When the abdomen was cut into, a large tumour presented itself, occupying the whole of the lower portion. It was spherical, and measured about eight inches in its transverse diameter: in its diameter parallel with the body six inches, and it was four inches deep.

On examination, it was found to occupy the place of the left ovary. The fimbriated extremity of the right fallopian tube was attached to the right side of the tumor. The uterus, of the natural size and appearance, lay immediately below and behind it. The right ovary was slightly enlarged, and of a very dark colour.

On cutting into the tumour it was found to contain a cheese-like substance, with small masses of hair, quite compact, mixed through it. The amount of this matter was neither weighed nor measured; but it was supposed to be about three pints. The walls of the tumor were firm and resisting, and presented, on their internal surface, the appearance of the bones of a fœtus. One of the scapulæ and the parietal bones were, indeed, quite distinct. The mass was preserved in salt water for further inspection.

